# Primary care transformation in Scotland: a qualitative study of GPs’ and multidisciplinary team members’ views

**DOI:** 10.3399/BJGP.2023.0086

**Published:** 2023-12-29

**Authors:** Eddie Donaghy, Huayi Huang, David Henderson, Harry HX Wang, Bruce Guthrie, Stewart W Mercer

**Affiliations:** Usher Institute, College of Medicine and Veterinary Medicine, University of Edinburgh, Edinburgh, UK.; Usher Institute, College of Medicine and Veterinary Medicine, University of Edinburgh, Edinburgh, UK.; Usher Institute, College of Medicine and Veterinary Medicine, University of Edinburgh, Edinburgh, UK.; School of Public Health, Sun Yat-Sen University, Guangzhou, China.; Usher Institute, College of Medicine and Veterinary Medicine, University of Edinburgh, Edinburgh, UK.; Usher Institute, College of Medicine and Veterinary Medicine, University of Edinburgh, Edinburgh, UK.

**Keywords:** GP contract, health inequalities, multidisciplinary working, multimorbidity, primary care, primary care transformation, reform, rurality

## Abstract

**Background:**

The Scottish Government’s vision to transform primary care includes expansion of the primary care multidisciplinary team (MDT), formalised in the new GP contract in April 2018.

**Aim:**

To explore practitioners’ views on the expansion of MDT working in Scotland.

**Design and setting:**

Qualitative study with GPs and a range of MDT staff working in three different population settings in Scotland.

**Method:**

In-depth semi-structured interviews were carried out by telephone with 8 GPs and 19 MDT staff between May and June 2022. Interviews were audio-recorded and transcribed verbatim. Thematic analysis was conducted to identify commonalities and divergences in the interviews.

**Results:**

Internal challenges facing MDT staff included adapting to the fast pace of primary care, building new relationships, training and professional development needs, line management issues, and monitoring and evaluation of performance. External challenges included the ongoing effects of the COVID-19 pandemic, lack of time, difficulties with hybrid working, and low staff morale. Most GPs reported that expansion of their roles as expert medical specialists had not yet happened because their workload had not decreased (and in many cases had increased). In deprived areas, insufficient resources to deal with the high numbers of patients with complex multimorbidity remained a key issue. Interviewees in remote and rural settings felt the new contract did not take into account the unique challenges of providing primary care services in such areas, and recruitment and accommodation were cited as particular problems.

**Conclusion:**

Although there has been substantial expansion of the primary care MDT, which most GPs welcome, many challenges to effective implementation remain that must be addressed if transformation of primary care in Scotland is to become a reality.

## Introduction

In response to an ageing population, growing numbers of people living with multiple long-term conditions (multimorbidity), and widening health inequalities, the Scottish Government has a policy vision of better integration of care, and shifting the balance of care from hospital to community.^1^^,^^2^ Consequently, the transformation of primary care is seen as fundamental to achieving better-quality patient care, delivering effective management of multimorbidity, and reducing or mitigating health inequalities while also reducing costs.^3^^–^^5^

Expansion of the general practice workforce is fundamental to the Scottish Government’s aim of moving more care into the community and, in December 2017, it pledged to increase GP numbers by 800 within a decade.^6^ However, an ageing GP workforce, and GP recruitment and retention problems, have made it difficult for the Scottish Government to meet its target.^7^^,^^8^ Between 2018 and 2021, GP numbers in Scotland increased by 209, but this was solely because of an increase in female GPs, who mostly work part-time.^9^ The most recent data published in November 2022 showed a 3% fall in the number of full-time GPs in Scotland, the lowest level since 2009.^10^

To address problems with GP recruitment and retention, and to facilitate primary care transformation, in April 2018 a new General Medical Services contract was introduced in Scotland.^11^^,^^12^ The contract aimed to reduce GP workload and refocus the role of GPs towards becoming expert medical generalists, allowing them to focus more of their time on patients with complex care needs such as people living with multimorbidity.^11^^,^^12^ A key part of the new contract is the expansion of the multidisciplinary team (MDT) in primary care so that patients can receive care from the most appropriate member of the MDT and at the right time and place with the intention of also helping to reduce GP workload.

Wider primary care transformation in Scotland has also seen the integration of health and social care,^13^ and in 2016 the introduction of GP clusters (*‘typically groups of between five to eight GP practices in a close geographical location’*),^14^ which the authors have previously reported on.^15^^–^^17^ Between March 2018 and March 2022, the Scottish Government reported that there were 3220 whole-time equivalent new MDT staff appointed in primary care, with the largest group being pharmacists and pharmacy technicians (just under 1000).^18^

**Table:** How this fits in

The Scottish Government introduced expansion of multidisciplinary teams (MDTs) as part of its vision to transform primary care in early 2016, formalised in a new Scottish GP contract in April 2018. These changes aimed to reduce GPs’ workload, improve patient care, and better meet the needs of patients with complex problems. This qualitative study with GPs and MDT staff reveals that, although most GPs welcomed the expansion of the MDT, there are many internal and external challenges to the effective implementation of integrated MDT working in primary care in Scotland. Most GPs reported that their workload had not decreased (and in many cases had increased) and the care of patients with complex multimorbidity had not improved. Challenges were most marked in deprived areas and in remote and rural settings. These barriers to effective MDT working need to be addressed if the ambitions of the new GP contract and the transformation of primary care in Scotland are to be fulfilled.

The aim of this study was to explore the views of GPs and MDT staff on these factors, and the wider progress of primary care transformation (and its impact on ageing people with multimorbidities and on health inequalities) in three different health board areas of Scotland (urban high deprivation, urban mixed affluent/ deprived, and remote and rural).

## Method

This research is presented using the standards for reporting qualitative research framework.^19^ The research was carried out and reported in accordance with the consolidated criteria for reporting qualitative research.^20^

### Study design

Qualitative methods were used to explore the views of frontline primary care staff working in Scottish general practices. Data were collected using in-depth semi-structured interviews. As a result of COVID-19, all interviews were conducted by telephone, something commonly done by researchers during the pandemic.^21^

### Sampling and recruitment

This study is the second phase of an ongoing programme of funded research led by the corresponding author on primary care transformation in Scotland. As part of this broader programme, 12 GP clusters were recruited across three Scottish health boards (out of a total of 14) for qualitative and quantitative evaluation. This number of health boards was established by the funding limit of the research grant. The participating health boards were selected to provide a variety of population characteristics, including urban areas of high deprivation (health board 1), urban mixed affluent/ deprived (health board 2), and remote and rural populations (health board 3). Frontline primary care staff working in GP practices across participating health boards and 12 participating GP clusters were initially approached by cluster quality leads who had participated in phase 1 of the qualitative data collection.^17^ Potential participants were provided with details of the research through a participant information sheet. The research team was accessible to answer any questions about the study. Recruitment stopped when the core research team agreed that data saturation had been reached. The health boards and the participating GP clusters and practices are not named to preserve the confidentiality of the participants.

### Data collection

One-to-one telephone interviews with frontline GP practice staff, lasting approximately 40–60 minutes, were conducted by two authors between May and June 2022. Interviews were audio-recorded and transcribed verbatim. The interview topic guide was influenced by phase 1 of the authors’ previous qualitative work in this current study, a scoping review of literature on primary care transformation in Organisation for Economic Co-operation and Development (OECD) countries. Areas covered with participants included their views on the original intentions of the GP contract/ reforms and expected outcomes at that time, their views on actual progress locally in the GP practices they worked in (particularly MDT expansion and its impact on GP workload and support and training for MDT staff), the impact on health inequalities and addressing the needs of older people living with multimorbidity, and the effect of COVID-19. Table 1 shows the wide range of ages, experience, and professional roles of the interviewees across the three health boards. The professional roles included covered most of the key professions involved in MDTs in primary care in Scotland.^18^

**Table 1.** Characteristics of participating frontline GPs and MDT staff (*n* = 27)

**Health board and participant interviewee number**	**Sex**	**Professional role**	**Years since qualified**	**Years in current post professional**	**Employer**	**Line manager**
**Health board 1, urban high deprivation (*n* = 9)**						

P03	Female	Senior prescribing support pharmacist	24	15	Health board	HSCP/Health board
P06	Female	Advanced nurse practitioner/practice nurse	25	<1	GP practice	GP practice
P11	Female	GP partner	19	5	Independent contractor and health board	Independent contractor and health board
P12	Male	Community links worker	N/A	<1	Third-sector organisation	Third-sector organisation
P15	Male	GP partner	19 in medicine, 9 as GP	8	GP practice	Independent contractor
P17	Male	Advanced physiotherapy practitioner	18	4	Health board	Health board
P18	Female	Advanced nurse practitioner	30	7	HSCP	HSCP
P20	Female	Community links worker	8	<1	Third-sector organisation	Third-sector organisation
P27	Male	GP	16	2	Independent contractor	Independent contractor

**Health board 2, urban affluent/mixed affluent/deprived (*n* = 10)**						

P01	Female	Specialist clinical pharmacist	6	1.5	Health board	Health board
P02	Female	Advanced nurse practitioner	20	2	Health board	Health board
P08	Female	Practice nurse	13	6	GP practice	GP practice
P10	Female	Physician associate	<1	<1	Health board	GP practice
P13	Female	Community links worker	N/A	2.5	Third-sector organisation	Third-sector organisation
P14	Male	GP partner	24	15	Independent contractor	Independent contractor
P19	Female	Advanced physiotherapy practitioner	19	3	Health board	Health board
P22	Female	Practice nurse	21.5	5	GP practice	GP practice
P23	Male	GP partner	8	7	Independent contractor	Independent contractor
P24	Female	Salaried GP	20 as doctor, 5 as GP	5	GP practice	GP partners

**Health board 3, remote and rural (*n* = 8)**						

P04	Female	Pharmacy team lead	21	<1	Health board	Health board
P05	Female	Pharmacy team lead	20	2.3	Health board	Health board
P07	Female	First-contact physiotherapist	10	2.5	Health board	Health board
P09	Female	First-contact physiotherapist	11	2	Health board	Health board
P16	Male	GP partner	4	1.5	Self-employed	Self-employed
P21	Female	GP partner	14	6	Self-employed	Self-employed
P25	Female	Practice nurse	32	21	GP practice	GP practice
P26	Female	Practice manager	N/A	17	GP practice	GP practice

*HSCP = Health and Social Care Partnership. MDT = multidisciplinary team.*

### Data analysis

Thematic content analysis was conducted^22^^,^^23^ to identify commonalities and divergences regarding the significance and operationalisation of the GP contract/wider reforms in the context of the three diverse population groups in each health board, and from the perspective of the different frontline professionals interviewed. Three authors independently identified and developed initial codes based on individual analysis of selected interview transcripts from the three health boards, and agreed on the coding frame through discussion. The transcripts were coded using NVivo Pro (version 12). The phases of thematic analysis outlined by Braun and Clarke^22^^,^^23^ were applied by the core team of researchers in the six following steps: familiarisation with the data, generation of initial codes, searching for themes, reviewing themes, defining and naming themes, and producing the final report. The agreed themes were also discussed with the wider research team, including the four members of the patient and public involvement group established for this programme of research, and sent to participants for comments. The key findings are reported below with examples of quotations. See Supplementary File for details of more quotations on all the themes identified.

## Results

Most interviewees spoke positively about an increase in MDT working, and reported that patients were seeing a range of MDT staff since the introduction of the new GP contract in Scotland:

*‘We’re quite far on in having other health professionals in the practice. We’ve got a physiotherapist, a pharmacist, a physician’s assistant. We’re signposting through reception a lot of our patients* directly to them at first point of contact.*’* P23

(GP, partner, male, urban mixed affluent/ deprived practice)

*‘It just feels like certain problems lend themselves really well to being managed by those AHPs* [allied health professionals] *as a first point of contact. That’s been a really positive change. On the days or the weeks in which those professionals are not available, we really notice the difference.’* P24

(GP, female, urban mixed affluent/deprived)

However, three key common themes were identified in the analysis in terms of challenges of MDT implementation: the intrinsic challenges of MDT expansion in primary care, the extrinsic challenges relating to the COVID-19 pandemic and post-pandemic period, and the impact on GP workload and the GP’s expert generalist role. Issues were also identified that differed between the three sites.

### Theme 1: Intrinsic challenges of MDT expansion

Theme 1 had five sub-themes: adjusting to the primary care setting, building relationships, training and professional development, line management, and monitoring and evaluation.

#### Adjusting to the primary care setting

MDT staff new to primary care reported challenges adjusting to the setting, including higher patient volumes, greater patient complexity and clinical uncertainty, shorter appointment times, the unique cultures of individual GP practices, isolation, and working across different GP practices:

*‘With these roles it’s shorter appointments and high-volume patients. We’re seeing far more in primary care than in secondary … The biggest stressor of the job is the volume of patients and the unpredictable day … there is that risk of burnout I suspect.’* P17

(advanced physiotherapy practitioner, male, urban deprived)

*‘Coming from secondary care into the community, for me, was very difficult. The differences are absolutely huge. It is a completely different mindset. You don’t have the answers in front of you … It’s navigating the hospital without walls … Loneliness is an issue. It’s a very independent role. You’re the sole decision maker. You’re not on a ward round, you’re in somebody’s house, or sitting in a clinic room on your own.’* P02

(advanced nurse practitioner, female, urban mixed affluent/deprived)

#### Building relationships

MDT staff reported positive and negative experiences of building relationships within the practices:

*‘I think our MDT roles are very well supported here … I speak to the GPs very regularly. They are very approachable … It makes me feel very much part of the team. It has been very welcoming and very supportive. However, I know that that is not the case in every practice.’* P19

(GP partner, male, advanced physiotherapy practitioner, urban mixed/affluent/ deprived)

*‘When we initially start in a practice it can be quite overwhelming. You might feel a bit lonely as I did. So, it was stepping out of my comfort zone, making the effort to go and speak to people, go to tea and lunch breaks … It’s taken some time to integrate into the team.’* P01

(specialist clinical pharmacist, female, urban mixed/ affluent/deprived)

The importance of shared expectations around roles and responsibilities was also regarded as important in relationship building:

*‘It’s very variable in the GP practices how they look at us and what work they send us. In some practices we work really well as a team. In one practice we’ve got a reasonable amount of work. Time is set aside for medication reviews, cost-efficiencies, that’s agreed. But in other practices that doesn’t happen and the sheer volume of work isn’t sustainable. They try and offload the work to us. So the expectations from the GPs about our workload aren’t realistic.’* P03

(senior prescribing support pharmacist, female, urban deprived)

MDT staff also highlighted the importance of trust in relationship building:

*‘So one of the challenges is moving to a new GP practice team. You have to really make your own mark in that team, and gain that trust. If you don’t have it already, it’s a huge challenge … As a team, if you have trust then you’ll get there, and if you don’t, then you won’t.’* P02

(advanced nurse practitioner, female, urban mixed affluent/deprived)

#### Training and professional development

Although MDT staff welcomed further developing their professional roles and skills, some raised questions about levels of training and professional development opportunities:

*‘More support is desperately needed for staff as they roll out … They’re thrown into these roles … If I was to stand up in a court of law: “Can you justify why you did this and that and what training you had to do this?” I’d be a little bit concerned.’* P17

(advanced physiotherapy practitioner, male, urban deprived)

#### Line management

Line management was felt to be problematic by several of those interviewed, as MDT staff were practice based but often employed and line managed by the Health and Social Care Partnership (HSCP):

*‘If we have an ANP* [advanced nurse practitioner] *and we’re told by the HSCP “Well, they don’t work after one o’clock on a Friday.” You don’t have control over that … But then that’s the problem if they’re being line managed by somebody else. You’re working for two teams.’* P11

(GP partner, female, urban deprived)

However, one ANP saw being employed by the HSCP as a safety net:

*‘The difficulty lies obviously in GPs aren’t employed by the HSCP … As ANPs, I think we’re safeguarded because we’ve got the HSCP behind us. The HSCP come into bat for us if they don’t think we’re being treated fairly by a GP practice.’* P18

(ANP, female, urban deprived)

#### Monitoring and evaluation

A number of interviewees raised the issue of how to assess progress and the lack of monitoring, evaluation, feedback, and time to reflect:

*‘I think you have to be careful that you’re not just churning through volumes and volumes of patients. That you get time to reflect … to have non-clinical time to develop the service … I think we can anecdotally say what we’re doing, but perhaps not have the evidence to back that up.’* P17

(advanced physiotherapy practitioner, male, urban deprived)

*‘There isn’t enough time … you’re fire-fighting rather than spending time planning and bringing people together work-wise … It’s time to start thinking “What do we do going forward?” … We’re not consistent at auditing the changes and then reinforcing change.’* P08

(practice nurse, female, urban mixed/affluent/ deprived)

### Theme 2: Extrinsic challenges relating to the COVID-19 pandemic and post-pandemic period

#### The pandemic

Interviewees reported that the COVID-19 pandemic had a very large negative effect on the progress of the new GP contract and the MDT:

*‘COVID stopped everything. In terms of long-term conditions management, a lot of it stopped.’* P05

(pharmacy team lead, female, remote and rural)

Most interviewees also reported increased workloads post-pandemic due to backlogs and patient demand:

*‘COVID has made things a lot less efficient and particularly post-COVID we’ve got this huge backlog of clinical need that’s built up.’* P14

(GP partner, male, urban mixed affluent/deprived)

Added to this was also an increase in the complexity of patients’ needs:

*‘We’re now seeing people coming forward who are sicker than they might have been … there’s a lot of people who are really sick at the minute with big problems.’* P26

(practice manager, female, remote and rural)

#### Time pressure

Unsurprisingly, time pressure was a key issue:

*‘Enough time? I very rarely get a lunchbreak and I’m always late after work. So, no, definitely. No, you’re always looking at the clock. No, it’s quite clinical based at the moment because everyone’s just under pressure.’* P09

(first-contact physiotherapist, female, remote and rural)

#### Hybrid working

A number of interviewees believed that hybrid consulting, introduced during the pandemic as a necessity, would remain in place:

*‘I would say about thirty to forty per cent are face-to-face appointments. It’s only by making phone calls that I can get through the number of patients that need to be seen … Hybrid working is here to stay.’* P15

(GP partner, male, urban deprived)

However, many felt that hybrid working carried significant risks:

*‘Telephone triage has become the new norm which incorporates much higher risk. Quite often we will say at our coffee meetings “I saw this, it sounded nothing like that on the phone. I’m so glad I brought them in and saw them.” We’ve had some near misses, we’ve had some delayed diagnoses from that.’* P23

(GP partner, male, urban mixed affluent/deprived)

#### Staff morale

Most interviewees reported damage to staff morale during and since the pandemic:

*‘Morale is very low at the moment, just kind of feels like you’re not ever going to get out of this and get back to normality. I think people have lost any resilience they had.’* P03

(senior prescribing support pharmacist, female, urban deprived)

### Theme 3: Impact on GP workload and the GP’s expert generalist role

Seven of the eight GPs interviewed reported no decrease in workload, and most reported increased workload post-pandemic. Consequently, most GPs had not been able to give targeted longer consultations to patients with complex needs:

*‘Am I spending more time as an expert medical generalist? No. Because there’s nobody that can actually take the work off us. I don’t have longer appointment times … Patient demand, it’s doubled in the past couple of years, since COVID.’* P15

(GP partner, male, urban deprived)

GPs also spoke about the increased responsibilities they have in supervising and training MDT staff:

*‘A lot of our time as GPs will be supervising AHPs … To train and develop autonomous working in AHPs takes a long time … Time is precious. You often don’t feel as though you have the time to be able to properly input and teach them.’* P23

(GP partner, male, urban mixed affluent/deprived)

#### Health inequalities

Most interviewees believed the new contract had done little to reduce health inequalities; indeed, several noted that health inequalities had widened during and following the COVID-19 pandemic:

*‘The impact of COVID on health inequalities? It’s widened that further. A good example is that there was a map disseminated in the first lockdown of all the hotspots of where the most prevalent areas of COVID were, they were all the most deprived areas. So that’s just a huge sign of health inequalities. These are the people getting COVID, where the people with chronic health diseases are most vulnerable and more of them are getting it in these areas. So, COVID definitely has emphasised and worsened that further.’* P27

(GP, partner, male, urban deprived)

### Differences between affluent/ mixed, deprived, and remote and rural settings

#### Deprived

In the deprived areas, interviewees more commonly reported insufficient resources to deal with the high numbers of patients with complex multimorbidity (spanning mental, physical, and social problems):

*‘In areas of high deprivation people have multiple medical problems, social and mental health issues … It’s a challenging demographic, trying to encourage self-management, and health promotion and wellbeing … Also it’s still a hard sell getting patients used to not seeing a GP … In the more affluent practice area, patients are coming in primarily with single musculoskeletal problems.’* P17

(advanced physiotherapy practitioner, male, urban deprived)

Interviewees reported that the pandemic had exacerbated health inequalities, and staff morale was lower in these interviewees compared with those from more affluent or remote and rural settings.

#### Urban mixed affluent/deprived

Interviewees from the urban affluent/mixed health board more often had a positive view of the new contract’s impact:

*‘There is not a lot of socially deprived areas round here. Generally, patients are very health literate … A lot of people will have private health care as well, so the referrals into private health care I can do as well … and I think there is a really good balance of staff and an appropriate balance of MDT staff.’* P19

(advanced physiotherapy practitioner, female, urban mixed affluent/deprived)

#### Remote and rural

Interviewees in this setting generally felt that the new contract did not take into account the challenges of providing primary care services in remote and rural areas. Recruitment and affordable accommodation were cited as particular problems:

*‘If you’re looking at the central belt, if it’s a pharmacist they maybe go to that practice every day. But for us, that doesn’t make sense, with the travel distance involved because it’s two hours away. That’s something that isn’t in the contract, and it’s not been decided where we should work from … The contract is very much city centric, sadly … We’ve got some increased MDT working, but it’s limited. The issue is around recruitment and retention in rural areas. Accommodation is also a serious issue.’* P05

(pharmacy team lead, female, remote and rural)

## Discussion

### Summary

This study involving GPs and MDT staff reveals many internal and external challenges to the expansion and implementation of integrated MDT working in primary care in Scotland. Internal challenges facing MDT staff included adapting to the fast pace of primary care, building new relationships, training and professional development needs, line management issues, and monitoring and evaluation of performance. External challenges included the ongoing effects of the COVID-19 pandemic, lack of time, difficulties with hybrid working, and low staff morale. Most GPs reported that their workload had not decreased (and in many cases had increased) and the care of patients with complex multimorbidity had not improved. Challenges were most marked (although different) in deprived areas and in remote and rural settings.

The introduction of growing numbers of MDT staff working in general practice, but not employed or line managed by senior practice staff, has clearly introduced a new dynamic into Scottish general practice. The findings of this study indicate that tensions can arise as a result of these new line management processes, especially where there are competing priorities between individual GP practices and health board and HSCP priorities. Although there has been substantial expansion of the primary care MDT, many challenges to effective implementation remain, which must be addressed if transformation of primary care in Scotland is to become a reality.

### Strengths and limitations

A strength of this study was the involvement of the four members of the patient and public involvement group in all stages of the study. A further strength was that clusters were purposively sampled in three health boards, in clusters serving urban deprived areas, urban mixed affluent/deprived areas, and remote and rural areas, in order to gain the views of GPs and MDT staff working in these diverse settings. However, as the first 12 clusters that agreed to participate in the research programme were recruited, it may have introduced a bias, in that those who volunteered to participate may have held stronger views, either positive or negative, than GPs and MDT staff as a whole. A further limitation was that not all types of MDT staff were recruited although most were covered; paramedics, mental health nurses, and healthcare assistants were not included in the sample as they did not volunteer to take part.^18^ Finally, as in all qualitative research, the findings of this study cannot be assumed to be generalisable.

### Comparison with existing literature

This study raises questions about the general integration of new MDT staff into primary care and how they adapt to the unique demands of general practice and individual practice contexts and culture. It also suggests that co-location of new MDT staff into GP practices in itself does not automatically ensure their integration or result in positive interdisciplinary working relationships, as other research has highlighted.^24^^–^^26^

The findings of the current study indicate that efforts to consciously build professional relationships and facilitate the best conditions for new MDT healthcare professionals to work effectively in GP practice teams are essential. This requires leadership at the practice level, fostering a practice culture that encourages integration and shared understanding of roles and responsibilities, building trust, good communication, and recognising the importance of individual GP practice culture and context, as others have highlighted.^27^^–^^30^ Recent research has likewise also noted that, to realise the potential of MDTs, local implementation needs to be carefully planned and supported by ongoing monitoring and evaluation.^31^

The importance of training and professional development was raised by participants, especially for MDT staff new to general practice and working in new general practice roles; research elsewhere also suggests that a lack of training and professional development can be a barrier to primary care transformation.^32^^,^^33^

Consequently, acknowledging the importance of building relationships and having processes in place to support this are fundamental for the development of effective multidisciplinary working in general practice. These two components are intertwined, as building positive working relationships can only be developed if the right processes are in place to facilitate them, along with shared understandings and agreements on MDT roles and responsibilities, as noted elsewhere.^29^^,^^30^ However, this remains problematic, as highlighted with, but not limited to, the integration of pharmacists into general practice,^34^^–^^36^ something also found in the current study. Our findings also agree with studies on the increased number of pharmacists working in GP surgeries that report challenges related to the working environment, as well as available support around training and time allowed for learning ‘on-the job’ to understand how primary care works. These studies concluded that, if integration of pharmacists into general practice is to be successful, flexibility is required to develop their roles based on individual general practice needs while performing within a recognised competency framework.

### Implications for practice

Introducing changes to general practice is challenging and policies aimed at transforming primary care frequently underestimate the time required from clinicians who already have very large workloads to take time out to support the implementation of policy goals.^37^^,^^38^ Doing so in light of the unprecedented challenges facing Scottish general practice as it emerges from the COVID-19 pandemic, as GP leaders in Scotland have highlighted,^39^ makes this a major test. A recent Scottish British Medical Association survey found that 81% of GP practices that responded to the survey reported that demand is exceeding capacity, underlining the scale of the challenge.^40^

While it is not possible to generalise the findings from such a small number of GPs interviewed, the feedback from the GPs who participated suggests that, while the introduction of more MDT staff into Scottish general practice has increased MDT working, it has not reduced GP workload. Indeed, many MDT staff interviewed corroborated this. With the increased responsibilities on GPs for training and supervision of new staff it has, in the short term at least, increased time pressures on GPs, echoing similar findings in England.^41^^,^^42^

Most interviewees believed that the new GP contract and wider reforms to transform primary care have had little impact on the care of older people living with multimorbidity or in addressing health inequalities. As recently highlighted by others,^43^^,^^44^ most interviewees, especially those working in deprived areas, believed the COVID-19 pandemic has exacerbated health inequalities.

Concerns over growing unmet need as general practice emerges out of the pandemic were raised by many interviewees, especially in deprived practices, suggesting a need for urgent answers to the inverse care law that continues to exist in Scottish general practice.^45^ The growing concerns of remote and rural GPs regarding the suitability and applicability of the GP contract where they practice,^46^ along with the growing problems of recruiting and retaining sufficient numbers of MDT staff, must also be considered by the Scottish Government.
